# Combined effects of vitamin D deficiency and systemic inflammation on all-cause mortality and cause-specific mortality in older adults

**DOI:** 10.1186/s12877-024-04706-x

**Published:** 2024-02-01

**Authors:** Chi Zhang, Ju Cui, Shaojie Li, Ji Shen, Xuanmei Luo, Yao Yao, Hong Shi

**Affiliations:** 1 The Key Laboratory of Geriatrics, Beijing Institute of Geriatrics, Institute of Geriatric Medicine, Beijing Hospital, Chinese Academy of Medical Sciences, National Center of Gerontology of National Health Commission, 100730 Beijing, China; 2 China Center for Health Development Studies, National School of Development, Peking University, Haidian District, 100191 Beijing, China; 3 Department of Geriatrics, Beijing Hospital, National Center of Gerontology, Institute of Geriatric Medicine, Chinese Academy of Medical Sciences, Dongcheng District, 100730 Beijing, China

**Keywords:** 25-hydroxyvitamin D, C-reactive protein, Mortality, Older adults

## Abstract

**Background:**

Vitamin D deficiency and systemic inflammation share common pathological mechanisms in muscle loss, cardio-pulmonary function decline, and abnormal metabolism, which are linked to chronic conditions, senescence, and early mortality. However, their combined effect on mortality in older adults has not been well established. This study longitudinal aimed to explore the independent and combined associations of serum 25-hydroxyvitamin D [25(OH)D] and high sensitivity C-reactive protein (hs-CRP) with mortality risk in Chinese community-based older people.

**Methods:**

3072 older adults (86.07 ± 11.87 years, 54.52% female) from the Chinese Longitudinal Healthy Longevity Survey (2012–2018) were enrolled. Baseline 25(OH)D and hs-CRP levels were collected, and survival information was recorded in the 2014 and 2018 follow-up waves. Cox proportional hazard regressions were conducted to explore the associations between 25(OH)D, hs-CRP, and mortality. Demographic characteristics, health behaviors, and chronic disease biomarkers were adjusted.

**Results:**

During 10,622.3 person-years of follow-up (median: 3.51 years), 1321 older adults died, including 448 deaths due to cardiovascular disease (CVD). Increased mortality risk was associated with lower 25(OH)D and higher hs-CRP quantiles, even after adjusting for each other and multiple covariates (all *P*-trend < 0.05). In combined analyses, the highest all-cause mortality (HR: 2.18, 95% CI: 1.73 ~ 2.56), CVD mortality (HR: 2.30, 95% CI: 1.64 ~ 3.21), and non-CVD mortality (HR: 2.19, 95% CI: 1.79 ~ 2.49) were obtained in participants with both 25(OH)D deficiency (< 50 nmol/L) and high hs-CRP (≥ 3.0 mg/L), respectively. We observed significant additive interactions of 25(OH)D and hs-CRP on all-cause mortality and non-CVD mortality (RERI_S_>0).

**Conclusions:**

Low 25(OH)D and high hs-CRP, both independently and jointly, increase mortality risk in Chinese community-dwelling older adults. Thus, priority should be given to early detection and appropriate intervention in older individuals with combined vitamin D deficiency and systemic inflammation. Molecular mechanisms of related adverse health effect are worthy of further investigation.

**Supplementary Information:**

The online version contains supplementary material available at 10.1186/s12877-024-04706-x.

## Background

Vitamin D is an important prominent micronutrient that is primarily involved in maintaining calcium homeostasis, which is mainly stored as 25(OH)D in the body [1]. Vitamin D deficiency has become a prevalent global health problem, particularly among individuals residing in developing nations, including China [2]. A recent meta-analysis of 472 research reports demonstrated that the prevalence of vitamin D deficiency, characterized by serum 25(OH)D level under 50 nmol/L, was 57.7% in the Asian demographic [3]. Associations of vitamin D levels and supplementation with mortality are not exactly consistent in published observational and intervention literatures though vitamin D has been linked to several potential health benefits [4–6]. Numerous studies have linked vitamin D deficiency with various negative health consequences, including fractures [7], frailty [8], sarcopenia [9], dementia [10], and premature death [11, 12]. Vitamin D may have anti-inflammatory properties and mitigate adverse systematic inflammation. Moreover, available literature also demonstrated that low levels of vitamin D exacerbated systemic inflammation during aging, potentially elevating the risk of several diseases as well as consequent mortality [13]. Nevertheless, the mechanisms underpinning the role of inflammation in the correlation between vitamin D and mortality has not been fully investigated. Inflammation is one of the crucial factors driving the process of human aging [14]. Review studies have reported strong associations between elevated inflammatory cytokines and multiple aging-related functional declines [15–17]. As an acute nonspecific biomarkers synthesized by hepatocytes, high-sensitivity C-reactive protein (hs-CRP) is widely used to reflect the degree of systemic inflammation [18]. Results from the NHANES showed that high hs-CRP was linked to increased all-cause mortality in older American population [19]. Moreover, previous cohort studies also confirmed that high hs-CRP was associated with mortality risk in Chinese and Korean older people [20, 21]. These findings suggested that vitamin D deficiency and elevated hs-CRP levels might be significant risk factors for mortality, yet few studies have focused on the combined impact of these two biomarkers.

Individual health outcomes are the result of a combination of factors, which are often interrelated [22]. A prior study has demonstrated a bidirectional relationship between lower vitamin D and elevated hs-CRP in older patients [23]. Besides, animal experiments also indicated that vitamin D deficiency and chronic inflammation might share common pathological mechanisms in muscle loss, cardio-pulmonary function decline, and abnormal metabolism, which are linked to chronic conditions, senescence, and early mortality [24–26]. These findings indicated that their coexistence might have a combined effect on mortality. Previous cohort studies have established the existence of interactions between inflammatory factors and 25(OH)D on the development of cardiovascular diseases [27], psychological disorders [28], and metabolic syndromes [29]. However, there is no substantial evidence to prove their combined association with mortality, particularly in Chinese community-dwelling older people. As the global population continues to age, China’s older population aged 65 years and above is estimated to reach 488 million by 2050 [30]. Thus, elucidating the effect of vitamin D deficiency and hs-CRP levels on mortality could be beneficial in preventing premature death and improving life expectancy.

To fill the gap in previous literatures, this study explored the independent and combined associations of 25(OH)D and hs-CRP on all-cause mortality and cause-specific mortality in community-dwelling older people based on the Chinese Longitudinal Healthy Longevity Survey (CLHLS).

## Methods

### Study population

We employed data from the CLHLS (2012–2018), an ongoing longitudinal study involving older adults from 23 provinces in China since 1998. Details of research design and sampling methods have been reported elsewhere [31]. Since 2008, the CLHLS has included sub-surveys in eight longevity regions (Xiayi County in Henan Province, Rudong County in Jiangsu Province, Yongfu County in Guangxi Province, Zhongxiang City in Hubei Province, Mayang County in Hunan Province, Chengmai County in Hainan Province, Laizhou City in Shandong province, and Sanshui District in Guangdong Province) to collect more comprehensive physical examinations and blood samples [32]. The current study included 3805 participants with complete baseline information and blood samples from the 2012 and 2014 biomarker sub-study waves. Participants below 60 years (*n* = 112), with missing data on serum25(OH)D or hs-CRP (*n* = 75), or more than 25% missing data on covariates (*n* = 25), were excluded. Those who were lost to follow-up for the first interview (*n* = 495) or had invalid death records (*n* = 26) were excluded due to inconclusive survival time. Hence, the final analysis included 3072 older individuals (86.07 ± 11.87 years, 54.53% female). The process of participant recruitment was displayed in Supplementary Fig. 1. The CLHLS was subjected to approval by the Ethics Committee of Peking University (No. IRB00001052-13074). All participants or their guardians provided written informed consent before the survey.

### Mortality and survival data

The survival status of participants was ascertained during the 2014 and 2018 follow-up surveys. The date of death and vital status were obtained from local official death certificates or participants’ next-of-kin. Survival time (in months) was derived as the duration between the first interview and the latest valid follow-up survey or death. Cardiovascular disease (CVD) mortality was recorded by the international classification of diseases (10th revision) codes of cardiovascular disease (I00-I78) or ischemic heart disease (I20-I25).

### Measurement of 25(OH)D

Fasting venous blood samples (5 mL) from all individuals who fasted overnight were collected by trained nurses and placed in a heparin anticoagulant vacuum tube. The serum samples were preserved at -80 °C after centrifugation at 2500 RPM. The concentration of 25(OH)D was measured by an enzyme-linked immunosorbent assay system (Bolton, UK) with the intra- and inter-assay variation less than 8% and 10%, respectively [33]. All laboratory tests were performed at the Capital Medical University in Beijing, and details of laboratory examinations have been published [34]. Vitamin D deficiency was defined according to the cutoff point (< 50 nmol/L) suggested by the Endocrine Society Guidelines [35]. We also divided 25(OH)D levels into quartiles to test the graded association between 25(OH)D and mortality risk.

### Measurement of hs-CRP

An automatic biochemistry analyzer (Hitachi 7180, Japan) was employed to determine the concentration of hs-CRP using immunoturbidimetric assay. The relevant quality control methods for laboratory examinations were described in previous study [36]. Consistent with previous studies, ≥ 3.0 mg/L was used as the cutoff value for high hs-CRP in the combined analyses [37]. The hs-CRP level was categorized as an ordinal variable (by quartiles) in the individual analyses.

### Covariates

The following covariates were included as potential confounders according to published literatures [21, 38–41]: age, sex, the month of sampling (as a continuous variable), the province of sampling, ethnicity (Han ethnic vs. minorities), residence (urban vs. rural), living status (living alone vs. with others), education levels (≥ 1 vs. <1 year of schooling), marital status (have no spouse including separated and widowed vs. current married), current smoking (yes vs. no), current drinking (yes vs. no), outdoor activities (frequently, occasionally, or rarely/never), vitamin product supplements (such as vitamin A/B/D/E: frequently, occasionally, or rarely/never), and body mass index (BMI). Medical care treatment was defined as receiving inpatient or outpatient medical services during the last year (yes vs. no). Physical activities of daily living (ADL) were evaluated by the Katz index (6 items); individuals with a total score < 6 points were designated as having ADL impairment. The Mini-Mental State Examination was used to assess participants’ cognitive function; illiterate subjects with an MMSE score of less than 18, or literate subjects with an MMSE score of less than 24 were defined as having cognitive impairment [42]. Depression was investigated using a single dichotomous question (In the past 2 weeks, did you feel sad or depressed?). Participants with systolic blood pressures ≥ 140 mmHg or diastolic blood pressures ≥ 90 mmHg, or those have been diagnosed by a doctor, were defined as having hypertension. Chronic disease-related biomarkers included albumin, fasting blood glucose, triglycerides, total cholesterol, and creatinine. Information on diagnosed cerebrovascular diseases, respiratory system diseases, and cancers was provided by the participants or their guardians through structured questionnaires.

### Statistical analysis

Descriptive data was presented as mean ± standard deviation, median (interquartile range), or numbers (percentages). Kaplan-Meier curves were conducted to compare the cumulative survival probability across different 25(OH)D and hs-CRP levels. Restricted cubic spline (RCS) regressions were applied to test the linear/nonlinear relationship between 25(OH)D, hs-CRP, and mortality risk. Three adjusted Cox proportional hazards models were applied to explore the independent and combined association of 25(OH)D and hs-CRP with mortality (Model 1, adjusted for age; Model 2, additionally adjusted for province of sampling, the month of sampling, ethnicity, residence, living status, education, marital status, outdoor activities, vitamin supplements, current smoking, current drinking, and BMI; Model 3, additionally adjusted for ADL, cognitive function, depression, albumin, blood glucose, cholesterol, triglycerides, creatinine, medical care treatment, hypertension, cerebrovascular disease, respiratory disease, and cancer). The assumption of proportional hazards was confirmed through Schoenfeld residuals tests. Hazard ratios (HRs) with 95% confidence intervals (CIs) were documented.

To analyze the combined effects of 25(OH)D and hs-CRP on mortality, a 4-level mutually-exclusive variable was created according to the presence of high hs-CRP and vitamin D deficiency in the fully adjusted Cox model (Group 1: vitamin D sufficiency + normal hs-CRP; Group 2: vitamin D deficiency alone; Group 3: high hs-CRP alone; Group 4: vitamin D deficiency + high hs-CRP). Participants with both vitamin D sufficiency and normal hs-CRP were designated as the reference group. According to the approach of Andersson et al. to biological interaction [43], the relative excess risk due to interaction (RERI) and 95% CIs were computed to evaluate the additive interaction between 25(OH)D and hs-CRP on mortality. When the RERI > 0 and 0 was not within its 95% CI, there was significant positive additive interaction [44]. The following sensitivity analyses were conducted to test the stability of primary results: (1) Since severe pathological conditions may confound the relationship, we sequentially excluded participants who suffered from cerebrovascular disease, respiratory disease, or cancer at baseline. (2) We performed a subgroup analysis based on whether the subjects had sought health care treatment within the past 1 year. (3) Considering the interference of outliers, participants with exorbitant measured values of 25(OH)D (≥ 100 nmol/L, *n* = 28) were excluded. To avoid acute infections or active autoimmune diseases, participants with hs-CRP ≥ 10 mg/L (*n* = 199) were also excluded to test the stability of main results. (4) Considering the potential confounding effect of vitamin supplementation, we excluded 169 older individuals who frequently supplemented with vitamins when exploring the association between 25(OH)D and mortality. (5) When analyzing the combined association between vitamin D and hs-CRP, we further adjusted for high-density lipoprotein and low-density lipoprotein in model 3. We used R software 4.2.0 version to perform all statistical analyses, and a two-tailed *P*-value < 0.05 indicated the significance level.

## Results

### Demographic characteristics

**Fig. 1:**
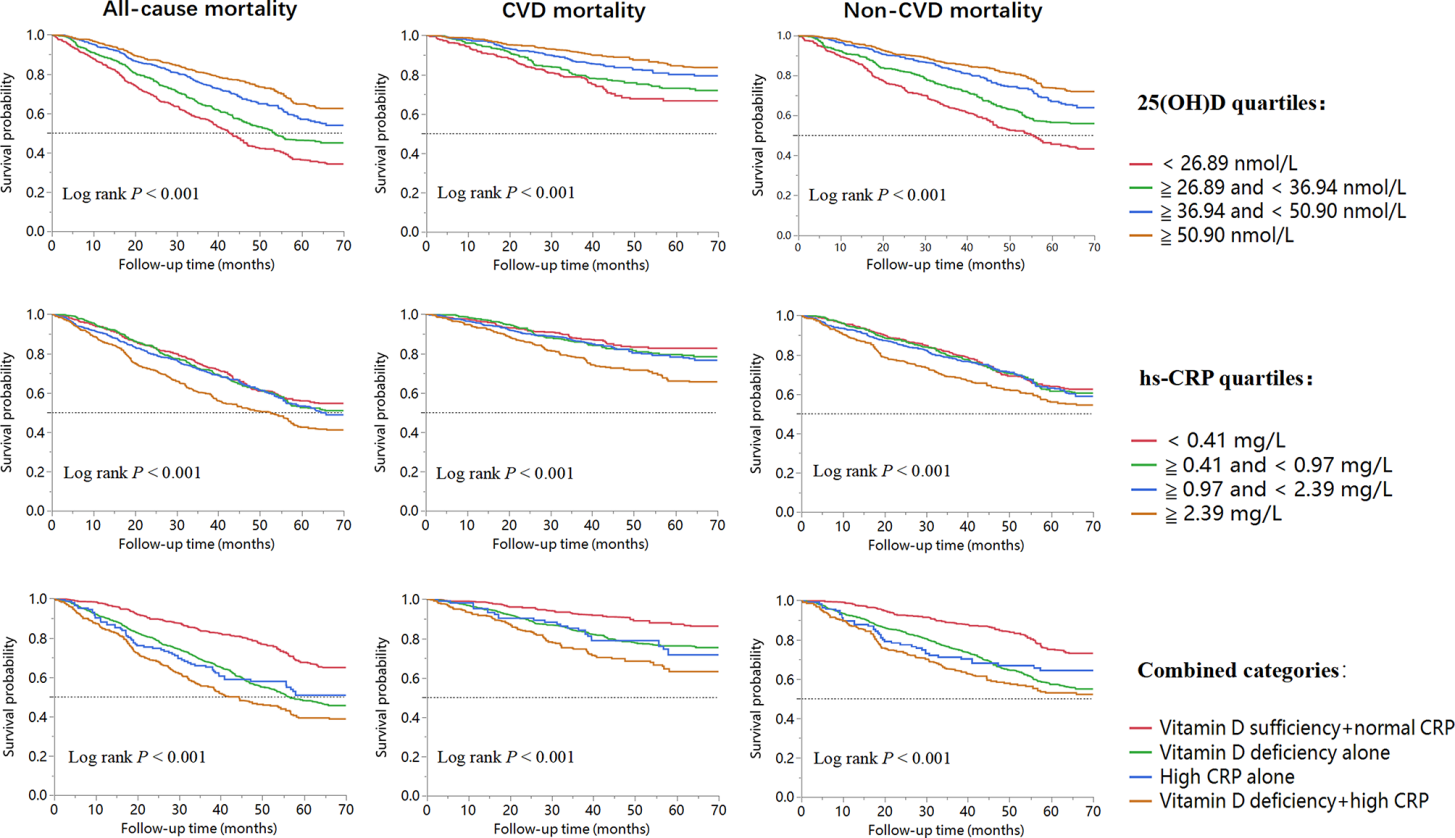
Kaplan-Meier survival curves for 3072 older adults stratified by 25(OH)D and hs-CRP levels

The mean age of 3072 older adults was 86.07 ± 11.87 years at baseline; among them, 1675 (54.52%) were female. Of all participants, 2258 (73.50%) had vitamin D deficiency, 644 (20.96%) had high hs-CRP, and 502 (16.34%) had both two conditions. Participants with both vitamin D deficiency and high hs-CRP were more likely to be older, illiterate, ADL impaired, cognitive impaired, had lower albumin, and had higher fasting blood glucose. Table 1 presented the demographics and clinical characteristics of all participants. During 10,622.3 person-years of the follow-up period (median 3.51 years), 1321 older adults died, including 448 CVD death events. Kaplan-Meier curves for all-cause, CVD, and non-CVD mortality across different 25(OH)D and hs-CRP quantiles were described in Fig. 1.

**Table 1:** Demographic characteristics of 3072 older adults across 25(OH)D and hs-CRP levels

Characteristics	Overall	Vitamin D sufficiency + normal hs-CRP	Vitamin D deficiency alone	High hs-CRP alone	Vitamin D deficiency + high hs-CRP	P value
Participants, n	3072	672	1756	142	502	
Mortality, n (%)	1321 (43.00)	185 (37.53)	799 (45.50)	65 (45.77)	272 (54.18)	< 0.001
**Socio-demographics**						
Age, year	86.07 ± 11.87	82.04 ± 12.11	86.92 ± 11.12	85.29 ± 11.75	88.74 ± 10.84	< 0.001
Female, n (%)	1675 (54.52)	237 (35.27)	1105 (62.93)	53 (37.32)	280 (55.78)	< 0.001
Time of sampling, month	6 (5,6)	6 (5,7)	6 (5,6)	6 (5,8)	6 (5,6)	< 0.001
Han ethnic, n (%)	2846 (92.64)	624 (92.86)	1643 (93.56)	128 (90.14)	451 (89.84)	0.033
Rural, n (%)	2502 (81.45)	580 (86.31)	1399 (79.67)	126 (88.73)	397 (79.08)	< 0.001
Live alone, n (%)	666 (21.68)	153 (22.77)	360 (20.50)	47 (33.10)	106 (21.12)	0.008
< 1-year schooling, n (%)	1984 (64.58)	341 (50.74)	1198 (68.22)	94 (66.20)	351 (69.92)	< 0.001
Have no spouse, n (%)	1823 (59.34)	308 (45.83)	1106 (62.98)	79 (55.63)	330 (65.74)	< 0.001
**Health status/behavior**						
BMI, kg/m^2^	21.49 ± 4.05	21.65 ± 3.65	21.46 ± 4.22	20.75 ± 3.37	21.61 ± 4.16	0.098
Outdoor activity, n (%)						0.083
frequently	1733 (56.41)	390 (58.04)	1000 (56.95)	75 (52.82)	268 (53.39)	
occasionally	197 (6.41)	55 (8.18)	106 (6.04)	10 (7.04)	26 (5.18)	
rarely or never	1142 (37.18)	227 (33.78)	650 (37.01)	57 (40.14)	208 (41.43)	
Vitamin supplements, n (%)						0.421
frequently	169 (5.50)	44 (6.55)	83 (4.73)	11 (7.75)	31 (6.18)	
occasionally	325 (10.58)	76 (11.31)	186 (10.59)	13 (9.15)	50 (9.96)	
rarely or never	2578 (83.92)	552 (82.14)	1487 (84.68)	118 (83.10)	421 (83.86)	
Current smoking, n (%)	481 (15.66)	156 (23.21)	232 (13.21)	28 (19.72)	65 (12.95)	< 0.001
Current drinking, n (%)	481 (15.66)	167 (24.85)	240 (13.67)	14 (9.86)	60 (11.95)	< 0.001
ADL impairment, n (%)	655 (21.32)	65 (9.67)	420 (23.92)	16 (11.27)	154 (30.68)	< 0.001
Cognitive impairment, n (%)	1224 (39.84)	188 (27.98)	743 (42.31)	48 (33.80)	245 (48.80)	< 0.001
Depression, n (%)	470 (15.30)	99 (14.73)	284 (16.18)	15 (10.56)	72 (14.34)	0.233
Medical care treatment, n (%)	1411 (45.93)	336 (50.00)	814 (46.36)	46 (32.39)	215 (42.83)	< 0.001
Hypertension, n (%)	1432 (47.48)	281 (41.94)	842 (48.06)	73 (51.77)	236 (47.48)	0.029
Cerebrovascular disease, n (%) (%)	249 (8.11)	53 (7.89)	141 (8.03)	7 (4.93)	48 (9.56)	0.304
Respiratory disease, n (%)	284 (9.24)	75 (11.16)	132 (7.52)	21 (14.79)	56 (11.16)	0.001
Cancer, n (%)	157 (5.11)	38 (5.65)	89 (5.07)	5 (3.52)	25 (4.98)	0.741
**Blood biomarkers**						
25(OH)D, nmol/L	40.22 ± 13.63	64.75 ± 14.55	31.51 ± 10.13	64.26 ± 14.56	30.93 ± 10.09	< 0.001
hs-CRP, mg/L	0.97 (0.41,2.39)	0.73 (0.35,1.37)	0.69 (0.34,1.28)	6.20 (4.03,11.71)	6.54 (3.98,14.21)	< 0.001
Albumin, g/L	41.40 ± 4.65	42.03 ± 4.33	41.10 ± 4.57	40.25 ± 4.73	39.27 ± 4.83	< 0.001
Blood glucose, mmol/L	4.69 (3.99,5.47)	4.66 (3.95,5.43)	4.67 (4.01,5.41)	4.61 (4.01,5.53)	4.87 (4.02,5.98)	0.013
Total cholesterol, mmol/L	4.48 ± 1.02	4.48 ± 0.91	4.53 ± 1.05	4.23 ± 0.86	4.36 ± 1.08	< 0.001
Triglycerides, mmol/L	0.91 (0.66,1.28)	0.89 (0.64,1.19)	0.92 (0.67,1.31)	0.86 (0.65,1.22)	0.93 (0.66,1.32)	0.061
Creatinine, mmol/L	81.41 ± 26.84	83.23 ± 22.13	78.29 ± 26.05	89.36 ± 26.67	87.63 ± 27.08	< 0.001

Continuous data were described as mean ± standard deviation or median (quartile: P_25_, P_75_)

*Notes*: ADL, activities of daily living; BMI, body mass index; 25 (OH)D, 25-dihydroxyvitamin D; hs-CRP, high sensitivity C-reactive protein. *P* values were generated using variance, Kruskal-Wallis, or chi-square test

### Independent associations of 25(OH)D and hs-CRP with mortality

**Fig. 2:**
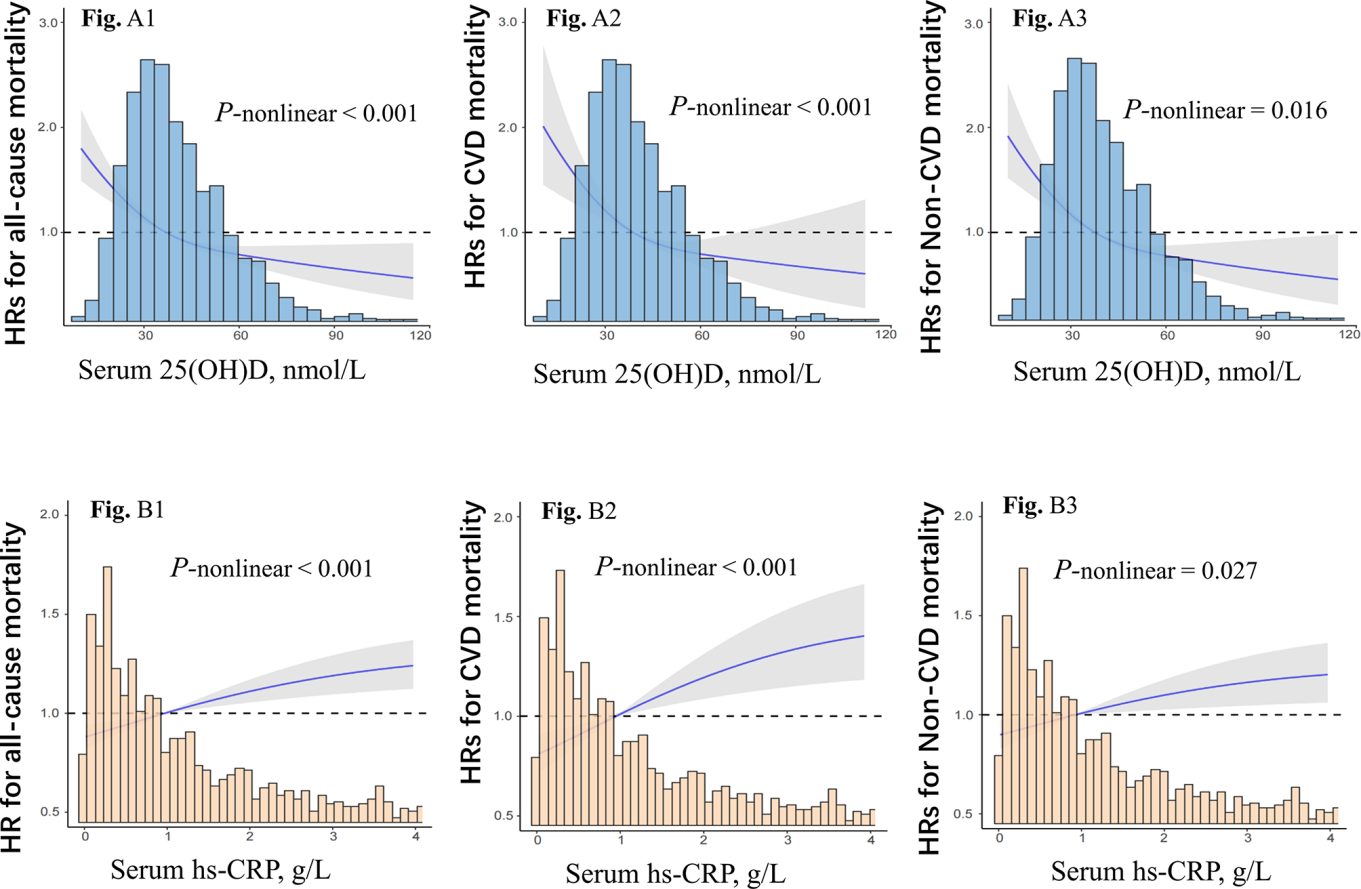
The restricted cubic splines for associations between 25(OH)D, hs-CRP, and mortality risk. Adjusting for age, sex, province of sampling, the month of sampling, ethnicity, residence, living status, education, marital status, outdoor activities, vitamin supplements, smoking, drinking, BMI, ADL impairment, cognitive function, depression, albumin, blood glucose, cholesterol, triglycerides, creatinine, medical care treatment, hypertension, cerebrovascular disease, respiratory disease, and cancer, 25(OH)D (in Fig. B1–B3), and hs-CRP (in Fig. A1–A3)

Using RCS regressions, we observed an L-shaped relationship of 25(OH)D and hs-CRP with all-cause, CVD, and non-CVD mortality (all *P*-nonlinear < 0.05) (Fig. 2). Descending curves indicated negative correlations between 25(OH)D and mortality (Fig2: A1–A3), while rising curves indicated positive correlation between hs-CRP and mortality (Fig2: B1–B3). Table 2 presented the HRs for mortality risk in three adjusted models. A graded association between increased mortality and lower 25(OH)D levels was observed after adjusting for age, sex, health status/behaviors, and multiple biological indicators (Model 3). This association remained significant even after considering the levels of hs-CRP (all *P*-trend < 0.05). Participants in the lowest 25(OH)D quartile showed a 55% increased risk for all-cause mortality (HR:1.55, 95%CI:1.30 ~ 1.85) and a 41% increased risk for CVD mortality (HR:1.41, 95%CI:1.05 ~ 1.93) compared with those in the highest quartile. Similarly, after adjusting for all covariates, higher hs-CRP was positively associated with increased all-cause mortality and cause-specific mortality (all *P*-trend < 0.05). Compared with the lowest hs-CRP quartile, the fully adjusted HRs of all-cause mortality and CVD mortality in the highest quartile group were 1.23 (95% CI: 1.05 ~ 1.44) and 1.35 (1.03 ~ 1.78), respectively.

**Table 2:** Independent associations of 25(OH)D and hs-CRP with mortality risk

Groups	Deaths, n%	HR (95% CI)				
		Model 1	Model 2	Model 3	Model 3 + hs-CRP	Model 3 + 25(OH)D
*Serum 25(OH)D*						
**All-cause mortality**						
≧ 50.90 nmol/L	236 (30.65)	Ref.	Ref.	Ref.	Ref.	
≧ 36.94 and < 50.90 nmol/L	284 (37.08)	1.27 (1.07 ~ 1.51)	1.30 (1.09 ~ 1.55)	1.20 (1.01 ~ 1.44)	1.20 (1.01 ~ 1.43)	
≧ 26.89 and < 36.94 nmol/L	369 (47.98)	1.59 (1.34 ~ 1.87)	1.59 (1.34 ~ 1.88)	1.36 (1.13 ~ 1.61)	1.34 (1.13 ~ 1.60)	
< 26.89 nmol/L	432 (56.32)	2.08 (1.77 ~ 2.45)	1.96 (1.65 ~ 2.32)	1.56 (1.31 ~ 1.86)	1.55 (1.30 ~ 1.85)	
*P*-trend		< 0.001	< 0.001	< 0.001	< 0.001	
**CVD mortality**						
≧ 50.90 nmol/L	82 (10.65)	Ref.	Ref.	Ref.	Ref.	
≧ 36.94 and < 50.90 nmol/L	100 (13.05)	1.32 (0.99 ~ 1.78)	1.31 (0.97 ~ 1.76)	1.25 (0.92 ~ 1.68)	1.24 (0.91 ~ 1.67)	
≧ 26.89 and < 36.94 nmol/L	130 (16.91)	1.67 (1.29 ~ 2.22)	1.64 (1.23 ~ 2.19)	1.38 (1.04 ~ 1.85)	1.37 (1.03 ~ 1.84)	
< 26.89 nmol/L	136 (17.73)	2.08 (1.56 ~ 2.75)	1.87 (1.39 ~ 2.53)	1.42 (1.06 ~ 1.93)	1.41 (1.05 ~ 1.93)	
*P*-trend		< 0.001	< 0.001	0.019	0.022	
**Non-CVD mortality**						
≧ 50.90 nmol/L	154 (20.00)	Ref.	Ref.	Ref.	Ref.	
≧ 36.94 and < 50.90 nmol/L	184 (24.02)	1.28 (1.03 ~ 1.58)	1.33 (1.07 ~ 1.64)	1.21 (0.96 ~ 1.50)	1.20 (0.96 ~ 1.50)	
≧ 26.89 and < 36.94 nmol/L	239 (31.08)	1.63 (1.33 ~ 2.01)	1.65 (1.34 ~ 2.03)	1.44 (1.17 ~ 1.78)	1.43 (1.16 ~ 1.78)	
< 26.89 nmol/L	296 (38.59)	2.27 (1.86 ~ 2.88)	2.21 (1.80 ~ 2.62)	1.82 (1.47 ~ 2.24)	1.80 (1.46 ~ 2.23)	
*P-*trend		< 0.001	< 0.001	< 0.001	< 0.001	
*Serum hs-CRP*						
**All-cause mortality**						
< 0.41 mg/L	293 (38.86)	Ref.	Ref.	Ref.		Ref.
≧ 0.41 and < 0.97 mg/L	319 (40.95)	1.03 (0.88 ~ 1.21)	1.04 (0.88 ~ 1.22)	1.00 (0.84 ~ 1.17)		1.00 (0.84 ~ 1.17)
≧ 0.97 and < 2.39 mg/L	315 (40.86)	1.09 (0.93 ~ 1.27)	1.08 (0.91 ~ 1.28)	1.08 (0.91 ~ 1.27)		1.08 (0.92 ~ 1.28)
≧ 2.39 mg/L	394 (51.30)	1.37 (1.18 ~ 1.59)	1.34 (1.14 ~ 1.57)	1.24 (1.06 ~ 1.45)		1.23 (1.05 ~ 1.44)
*P*-trend		< 0.001	< 0.001	0.004		0.005
**CVD mortality**						
< 0.41 mg/L	83 (11.01)	Ref.	Ref.	Ref.		Ref.
≧ 0.41 and < 0.97 mg/ L	104 (13.35)	1.14 (0.86 ~ 1.53)	1.15 (0.85 ~ 1.57)	1.05 (0.77 ~ 1.43)		1.05 (0.77 ~ 1.44)
≧ 0.97 and < 2.39 mg/L	106 (13.75)	1.22 (0.92 ~ 1.62)	1.17 (0.88 ~ 1.59)	1.09 (0.80 ~ 1.48)		1.07 (0.78 ~ 1.46)
≧ 2.39 mg/L	155 (20.18)	1.78 (1.37 ~ 2.33)	1.69 (1.28 ~ 2.23)	1.37 (1.04 ~ 1.85)		1.35 (1.03 ~ 1.78)
*P-*trend		< 0.001	< 0.001	0.021		0.032
**Non-CVD mortality**						
< 0.41 mg/L	210 (27.85)	Ref.	Ref.	Ref.		Ref.
≧ 0.41 and < 0.97 mg/L	215 (27.60)	1.01 (0.83 ~ 1.21)	1.01 (0.83 ~ 1.23)	0.97 (0.80 ~ 1.18)		0.97 (0.80 ~ 1.19)
≧ 0.97 and < 2.39 mg/L	209 (27.11)	1.05 (0.87 ~ 1.27)	1.06 (0.87 ~ 1.29)	1.09 (0.89 ~ 1.33)		1.10 (0.89 ~ 1.34)
≧ 2.39 mg/L	239 (31.12)	1.29 (1.07 ~ 1.55)	1.28 (1.06 ~ 1.54)	1.23 (1.02 ~ 1.50)		1.23 (1.02 ~ 1.49)
*P*-trend		0.007	0.013	0.014		0.015

Model 1: adjusted for age; model 2: additionally adjusted for sex, province of sampling, the month of sampling, ethnicity, residence, living status, education, marital status, outdoor activities, vitamin supplements, smoking, drinking, and BMI; Model 3: additionally adjusted for ADL, cognitive function, depression, albumin, blood glucose, cholesterol, triglycerides, creatinine, medical care treatment, hypertension, cerebrovascular disease, respiratory disease, and cancer. 25(OH)D, 25-dihydroxyvitamin D; hs-CRP, high sensitivity C-reactive protein; CVD, cardiovascular disease; HR, hazard ratios; CI, confidence interval

### Combined associations of 25(OH)D deficiency and high hs-CRP with mortality

Results of Cox regression analysis and RERIs were shown in Table 3. In the multiple-adjusted model, each group had a significant association with mortality relative to the reference group (vitamin D sufficiency + normal hs-CRP). The fully adjusted HRs of all-cause mortality for combined vitamin D deficiency and high hs-CRP levels was 2.18 (95% CI: 1.73–2.56), which was higher than that for deficiency of vitamin D alone (HR: 1.45, 95% CI: 1.16 ~ 1.62) and high hs-CRP alone (HR: 1.51, 95% CI: 1.14 ~ 2.03). Similarly, the highest CVD mortality (HR: 2.30, 95% CI: 1.64 ~ 3.21) and non-CVD mortality (HR: 2.19, 95% CI: 1.79 ~ 2.49) were obtained in participants with both two conditions. As Table 3 showed, significant positive additive interaction between vitamin D deficiency and high hs-CRP on all-cause mortality was observed (RERI: 0.22, 95% CI: 0.04 ~ 0.41) and non-CVD mortality (RERI: 0.21, 95% CI: 0.06 ~ 0.37), but not for CVD mortality (RERI: -0.36, 95% CI: -1.26 ~ 0.52). In the sex-stratified subgroup analyses, the relationship between 25(OH)D deficiency, high hs-CRP and mortality generally did not change appreciably. Besides, their combined effects were more pronounced in men (Table 3).

**Table 3:** Combined associations of 25(OH)D deficiency and high hs-CRP with mortality risk

Groups	Participants	All-cause mortality			CVD mortality			Non-CVD mortality		
		Deaths	Adj-HRs (95%CI)	P value	Deaths	Adj-HRs (95%CI)	P value	Deaths	Adj-HRs (95%CI)	P value
**Over all (*n*** = **3072)**										
Vitamin D sufficiency + normal hs-CRP	672	185	Ref.	—	60	Ref.	—	125	Ref.	—
Vitamin D deficiency alone	1756	799	1.45 (1.16 ~ 1.62)	< 0.001	255	1.76 (1.35 ~ 2.38)	0.001	544	1.47 (1.28 ~ 1.68)	< 0.001
High hs-CRP alone	142	65	1.51 (1.14 ~ 2.03)	< 0.001	26	1.90 (1.19 ~ 2.77)	0.004	39	1.50 (1.04 ~ 2.17)	0.031
Vitamin D deficiency + high hs-CRP	502	272	2.18 (1.73 ~ 2.56)	< 0.001	107	2.30 (1.64 ~ 3.21)	< 0.001	165	2.19 (1.79 ~ 2.49)	< 0.001
RERI (95%CI)			0.22 (0.04 ~ 0.41)			−0.36 (−1.26 ~ 0.52)			0.21 (0.06 ~ 0.37)	
**Men (*n*** = **1397)**										
Vitamin D sufficiency + normal hs-CRP	435	104	Ref.	—	32	Ref.	—	72	Ref.	—
Vitamin D deficiency alone	651	232	1.43 (1.06 ~ 1.70)	0.001	77	1.75 (1.15 ~ 2.71)	0.004	155	1.41 (1.06 ~ 1.79)	0.023
High hs-CRP alone	89	43	1.62 (1.11 ~ 2.29)	0.003	17	2.65 (1.38 ~ 4.27)	0.002	26	1.29 (0.82 ~ 1.96)	0.412
Vitamin D deficiency + high hs-CRP	222	119	2.25 (1.72 ~ 2.99)	< 0.001	45	2.91 (1.79 ~ 4.49)	< 0.001	74	2.51 (1.64 ~ 3.39)	< 0.001
RERI (95%CI)			0.19 (0.03 ~ 0.35)			−0.51 (−1.52 ~ 0.50)			0.80 (0.18 ~ 1.42)	
**Women (*n*** = **1675)**										
Vitamin D sufficiency + normal hs-CRP	237	81	Ref.	—	28	Ref.	—	53	Ref.	—
Vitamin D deficiency alone	1105	567	1.56 (1.23 ~ 1.97)	< 0.001	178	1.48 (0.97 ~ 2.21)	0.086	389	1.68 (1.17 ~ 2.41)	0.003
High hs-CRP alone	53	22	1.23 (0.81 ~ 1.95)	0.465	9	1.17 (0.66 ~ 2.53)	0.732	13	1.34 (0.84 ~ 2.21)	0.494
Vitamin D deficiency + high hs-CRP	280	153	1.87 (1.35 ~ 2.16)	0.005	62	1.84 (1.15 ~ 2.90)	0.021	91	1.99 (1.48 ~ 2.57)	0.008
RERI (95%CI)			0.07 (−0.23 ~ 0.38)			0.18 (−0.06 ~ 0.43)			−0.02 (−0.12 ~ 0.07)	

Adjusting for age, sex, province of sampling, the month of sampling, ethnicity, residence, living status, education, marital status, outdoor activities, vitamin supplements, smoking, drinking, BMI, ADL impairment, cognitive function, depression, albumin, blood glucose, cholesterol, triglycerides, creatinine, medical care treatment, hypertension, hypertension, cerebrovascular disease, respiratory disease, and cancer. 25(OH)D, 25-dihydroxyvitamin D; hs-CRP, high sensitivity C-reactive protein; CVD, cardiovascular disease; HR, hazard ratio; CI, confidence interval; RERI, relative excess risk due to interaction

### Sensitivity analyses

In sensitivity analyses, the main results were materially unchanged. Specifically, compared with the normal group, the HR of combined vitamin D deficiency/ high hs-CRP was still higher than that of these two conditions alone when participants with abnormal measurements of 25(OH)D or hs-CRP were excluded (Supplementary Tables 1 and 2). Consistent results were found after we sequentially excluded participants with cerebrovascular disease, respiratory disease, or cancer at baseline (Supplementary Table 3: panel A, B, and C), and significant positive additive interactions between vitamin D deficiency and high hs-CRP on all-cause mortality were also observed (RERIs > 0). Although monotonic and positive associations were observed in the two subgroups stratified by medical care treatment, we found that the effects of vitamin D deficiency and high hs-CRP on mortality risk were more pronounced in older adults who did not receive medical care in the last year (Supplementary Table 4). There was no significant interaction between medical care treatment and combined vitamin D deficiency/ high hs-CRP on all-cause mortality (*P*-interaction = 0.204), CVD mortality (*P*-interaction = 0.168), and non-CVD mortality (*P*-interaction = 0.239). The relationship between vitamin D levels and the risk of all-cause mortality did not change when we excluded participants who frequently supplemented with vitamins (Supplementary Table 5). In addition, additive interactions between vitamin D deficiency and high hs-CRP on all-cause mortality and non-CVD mortality were still significant when high-density lipoprotein and low-density lipoprotein were further considered (Supplementary Table 6).

## Discussion

This longitudinal study investigated the individual and combined associations of 25(OH)D and hs-CRP with mortality risk in Chinese older adults based on a 7-year prospective cohort. We revealed that low 25(OH)D and high hs-CRP jointly increased all-cause mortality and no-CVD mortality risk.

It was concluded that 25(OH)D deficiency alone was linked to higher mortality among older Chinese adults, which was consistent with most available literature [39, 40]. The UK Biobank research found a significant adverse association of 25(OH)D concentrations with all-cause, CVD, and cancer mortality [45]. A recent Chinese cohort study demonstrated a significant correlation between vitamin D changes and all-cause mortality [46]. However, another study of middle-aged older adults found no significant association between 25(OH)D and specific mortality [41]. These inconsistent findings from previous studies may be related to the differences in population characteristics. The distribution of serum 25(OH)D levels, dietary patterns, and the frequency outdoor activities in study samples may potentially influence the relationship between vitamin D and mortality. Previous studies in other countries and regions have also observed inverted J- or U-shaped relationships between 25(OH)D and mortality [6]. These results implied that population and ethnic heterogeneity might influence the association features, which must be confirmed by large cross-ethnic and population studies. We argued that the associations between vitamin D and specific mortality obtained in most observational studies might be due to the healthy sample in previous cohorts. Future RCTs should be conducted to support the effect of vitamin D supplementary on reducing mortality risk.

There are several mechanisms that link vitamin D deficiency to higher mortality including increased risk of cancer, cardiovascular disease, infections and fracture. Experimental researches have shown that the inadequate vitamin D was associated with secondary hyperparathyroidism and bone loss [47, 48]. This, in turn, increases the risk of falls and fractures [49], leading to injury-related deaths. Furthermore, certain studies have demonstrated that vitamin D could suppress adaptive immunity but promote innate immunity [50, 51]. Moreover, vitamin D deficiency is also linked to stronger autoimmunity as well as susceptibility to infection [52]. These studies indicate that older adults lacking adequate vitamin D are at a greater risk of developing autoimmune and infectious diseases, increasing the risk of death. In recent years, observational studies have confirmed the interaction of 25(OH)D and other biomarkers, such as albumin and interleukin-6 [39, 53], which might be a new approach to explain the complex association between vitamin D and multiple health outcomes in the future. Meanwhile, as for CVD mortality, serum 25(OH)D can regulate the renin-angiotensin system and the proliferation of vascular smooth muscle cells [54, 55], reducing the incidence of CVD diseases [56]. Besides, insufficient vitamin D was also found to be associated with depression, schizophrenia, and Alzheimer’s disease [12, 57]. The high prevalence of these diseases contributes to a significantly higher likelihood of future non-CVD mortality.

We also demonstrated that high hs-CRP was associated with increased all-cause, CVD, and non-CVD mortality. Similar findings were obtained from studies performed in the United States [19], Peru [38], and China [21]. A previous meta-analysis involving 22 studies showed significant positive relationships of hs-CRP with all-cause and CVD mortality [58]. Fundamental studies have also showed that hs-CRP could inhibit vascular endothelial growth factor-stimulated angiogenesis and indirectly promote vasoconstriction, thrombosis, and atherosclerosis [59, 60], increasing the risk of death from CVD [61]. Several cohort studies confirmed that hs-CRP was linked to the incidence and progression of cardiovascular and cerebrovascular events [62, 63]. High hs-CRP is also correlated with higher risks of various chronic disorders like diabetes [64], dementia [65], and cancer [66]. Furthermore, hs-CRP has been identified as a biomarker with poor prognosis for many diseases, such as cancer and myocardial infarction [67, 68]. These results suggest that high hs-CRP not only increases the risk of morbidity but also may lead to poor prognosis and thus increase the risk of death.

Although our findings on the separate association of 25(OH)D and hs-CRP with mortality were not novel, the innovative result was that vitamin D deficiency and high hs-CRP jointly increased risks of all-cause mortality by 118%, CVD mortality by 130%, and non-CVD mortality by 119%, which were higher than their individual effects. Significant additive interactions of 25(OH)D and hs-CRP on all-cause and non-CVD mortality were observed (RERI_S_>0), particularly in men. Some mechanisms may explain the combined association of vitamin D and hs-CRP with mortality. First, previous experimental studies have found that 25(OH)_2_D_3_, which is an active version of vitamin D, inhibits the synthesis of multiple inflammatory cytokines [69]. A population-based observational study also found that elevated circulatory 25(OH)D was significantly correlated with lower hs-CRP levels [70]. These results suggested that low serum 25(OH)D might contribute to high hs-CRP because of its anti-inflammatory properties. Therefore, vitamin D deficiency and high hs-CRP could aggravate each other, jointly increasing the risk of death. Second, vitamin D deficiency and high hs-CRP are included in common pathogenic factors of death which may ultimately increase the mortality risk. Previous studies have demonstrated that the coexistence of vitamin D deficiency and high hs-CRP was associated with a higher prevalence of sarcopenia [71, 72], CVD [27], schizophrenia [28], and metabolic syndrome [29]. Considering the potential confounding of clinical factors, we conducted multiple sensitivity analyses to test the stability of the main results. The combined associations of 25(OH)D and hs-CRP with mortality were materially unchanged after excluding participants with cerebrovascular disease, respiratory disease, or cancer at baseline. Interestingly, we found the effects of vitamin D deficiency and high hs-CRP on mortality risk were weaker in older adults who received inpatient or outpatient medical services in the last year (Supplementary Table 4). One possible reason was that vitamin D deficiency and inflammation might be treated with medical intervention when older people were cared for in medical facilities. Thus, we recommended focusing on the heterogeneity between clinical settings and healthy community samples in future studies.

### Strengths and limitations

This study focused on the interactions between multiple biomarkers and provides valuable epidemiological evidence. In the current study, 16.3% of older adults exhibited both vitamin D deficiency and high hs-CRP. Considering the combined association of vitamin D and hs-CRP with mortality, intervention strategies for maintaining adequate vitamin D and preventing inflammation may be beneficial in reducing mortality risk in community settings. The current study has several limitations. Firstly, while hs-CRP is a commonly used marker for systemic inflammation [73, 74], the present study did not account for other inflammatory factors, such as acute infections, autoimmune diseases, or anti-inflammatory drug use, etc. However, after excluding the outliers of hs-CRP measurements (≥ 10 mg/L), the main results remained stable, which may have partially eliminated the influence of immune-related diseases (Supplementary Table 2). Secondly, like other observational analyses, it is not possible to eliminate the residual confounding factors even after multivariable adjustment. Specifically, data on sunlight exposure was not available at baseline. To mitigate this limitation, we attempted to control for factors such as outdoor activity frequency, the month of the blood sampling, and residential provinces, following previous studies [39, 40]. Additionally, we were unable to quantify the supplementation of vitamin D. However, in this study, only a very small percentage (5.50%) of older individuals frequently supplemented with vitamins, which might not significantly confound the associations. Furthermore, when they were excluded, the relationship between vitamin D levels and the risk of mortality remained stable (Supplementary Table 5). Thirdly, we only measured serum 25(OH)D and hs-CRP levels from a single time point. Given that both may vary due to changes in lifestyle or health status, it is necessary to explore their dynamic effect on health consequences in in the future. Finally, despite the national representation of CLHLS, caution should be exercised when generalizing the findings to older people of other ethnicities.

## Conclusions

This study highlighted that the combination of low 25(OH)D and high hs-CRP levels could jointly elevate the risk of mortality in older adults. Thus, it is crucial to prioritize early detection and appropriate intervention for individuals who exhibit a combination of vitamin D deficiency and systemic inflammation.

### Electronic supplementary material

Below is the link to the electronic supplementary material.

Additional files: Supplementary Figure 1. Flowchart of participant recruitment and follow-up interviews. Supplementary Table 1. Sensitivity analysis of combined effects of 25(OH)D deficiency and high hs-CRP on mortality after excluding 26 participants with exorbitant measured values of serum 25(OH)D (≥100 nmol/L). Supplementary Table 2. Sensitivity analysis of combined effects of 25(OH)D deficiency and high hs-CRP on mortality after excluding 199 participants with exorbitant measured values of serum hs-CRP (≥10mg/L). Supplementary Table 3. Sensitivity analysis of combined effects of 25(OH)D deficiency and high hs-CRP on mortality after excluding participants with cerebrovascular disease, respiratory disease, or cancer at baseline. Supplementary Table 4. Subgroup analysis of combined effects of 25(OH)D deficiency and high hs-CRP on mortality stratified by medical care treatment. Supplementary Table 5. Associations of 25(OH)D with mortality risk after excluding 169 participants who frequently supplemented with vitamins. Supplementary Table 6. Combined associations of 25(OH)D deficiency and high hs-CRP with mortality risk after additionally adjusting for high-density lipoprotein and low-density lipoprotein.

## Data Availability

The raw data used in the current study can be found here: https://opendata.pku.edu.cn/dataverse/CHADS.

## Abbreviations

25(OH)D: 25-dihydroxyvitamin D
hs-CRP: high sensitivity C-reactive protein
CLHLS: Chinese Longitudinal Healthy Longevity Survey
CVD: cardiovascular disease
BMI: body mass index
ADL: activities of daily living
HR: hazard ratios
CI: confidence interval
