# Supplementary material for: Combined effects of vitamin D deficiency and systemic inflammation on all-cause mortality and cause-specific mortality in older adults
Source: BMC Geriatr. 2024 Feb 1;24:122. doi: 10.1186/s12877-024-04706-x (PMC10836043; doi:10.1186/s12877-024-04706-x)
Supplement: Supplementary file 1 — Additional files: Supplementary Figure 1. Flowchart of participant recruitment and follow-up interviews. Supplementary Table 1. Sensitivity analysis of combined effects of 25(OH)D deficiency and high hs-CRP on mortality after excluding 26 participants with exorbitant measured values of serum 25(OH)D (≥100 nmol/L). Supplementary Table 2. Sensitivity analysis of combined effects of 25(OH)D deficiency and high hs-CRP on mortality after excluding 199 participants with exorbitant measured values of serum hs-CRP (≥10mg/L). Supplementary Table 3. Sensitivity analysis of combined effects of 25(OH)D deficiency and high hs-CRP on mortality after excluding participants with cerebrovascular disease, respiratory disease, or cancer at baseline. Supplementary Table 4. Subgroup analysis of combined effects of 25(OH)D deficiency and high hs-CRP on mortality stratified by medical care treatment. Supplementary Table 5. Associations of 25(OH)D with mortality risk after excluding 169 participants who frequently supplemented with vitamins. Supplementary Table 6. Combined associations of 25(OH)D deficiency and high hs-CRP with mortality risk after additionally adjusting for high-density lipoprotein and low-density lipoprotein. [file 12877_2024_4706_MOESM1_ESM.docx]

**Supplementary materials**


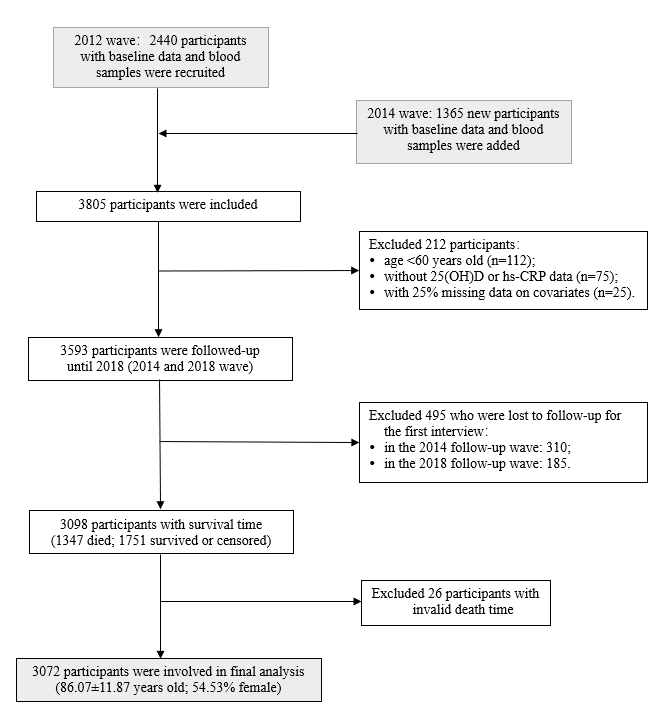


**Supplementary Figure 1. Flowchart of participant recruitment and follow-up interviews.**

**Supplementary Table 1. Sensitivity analysis of combined effects of 25(OH)D deficiency and high hs-CRP on mortality.**

**Excluding 26 participants with exorbitant measured values of serum 25(OH)D (≥100 nmol/L).**

| Groups | Participants | **All-cause mortality** | | | **CVD mortality** | | | **Non-CVD mortality** | | |
| --- | --- | --- | --- | --- | --- | --- | --- | --- | --- | --- |
|  |  | Deaths | Adj-HRs (95%CI) | *P* value | Deaths | Adj-HRs (95%CI) | *P* value | Deaths | Adj-HRs (95%CI) | *P* value |
| **25(OH)D <100nmol/L (n=3044)** |  |  |  |  |  |  |  |  |  |  |
| Vitamin D sufficiency + normal hs-CRP | 649 | 176 | Ref. | — | 55 | Ref. | — | 121 | Ref. | — |
| Vitamin D deficiency alone | 1756 | 799 | 1.53 (1.21~1.88) | <0.001 | 255 | 1.68 (1.23~2.28) | <0.001 | 544 | 1.52 (1.32~1.96) | <0.001 |
| High hs-CRP alone | 137 | 64 | 1.48 (1.11~1.97) | <0.001 | 26 | 1.85 (1.21~2.97) | <0.001 | 38 | 1.47 (1.03~2.14) | 0.029 |
| Vitamin D deficiency + high hs-CRP | 502 | 272 | 2.15 (1.68~2.49) | <0.001 | 107 | 2.36 (1.66~3.32) | <0.001 | 165 | 2.21 (1.71~2.85) | <0.001 |
| RERI (95%CI) |  |  | 0.13 (0.06~0.20) |  |  | -0.16 (-0.55~0.23) |  |  | 0.22 (0.05~0.39) |  |

Adjusting for age, sex, province of sampling, the month of sampling, ethnicity, residence, living status, education, marital status, outdoor activities, vitamin supplements, smoking, drinking, BMI, ADL impairment, cognitive function, depression, albumin, blood glucose, cholesterol, triglycerides, creatinine, medical care treatment, hypertension, cerebrovascular disease, respiratory disease, and cancer. 25(OH)D, 25-dihydroxyvitamin D; hs-CRP, high sensitivity C-reactive protein; CVD, cardiovascular disease; HR, hazard ratio; CI, confidence interval; RERI, relative excess risk due to interaction.

**Supplementary Table 2. Sensitivity analysis of combined effects of 25(OH)D deficiency and high hs-CRP on mortality.**

**Excluding 199 participants with exorbitant measured values of serum hs-CRP (≥10mg/L).**

| Groups | Participants | **All-cause mortality** | | | **CVD mortality** | | | **Non-CVD mortality** | | |
| --- | --- | --- | --- | --- | --- | --- | --- | --- | --- | --- |
|  |  | Deaths | Adj-HRs (95%CI) | *P* value | Deaths | Adj-HRs (95%CI) | *P* value | Deaths | Adj-HRs (95%CI) | *P* value |
| **CRP <10mg/L (n=2873)** |  |  |  |  |  |  |  |  |  |  |
| Vitamin D sufficiency + normal hs-CRP | 672 | 185 | Ref. | — | 60 | Ref. | — | 125 | Ref. | — |
| Vitamin D deficiency alone | 1756 | 799 | 1.56 (1.16~1.90) | <0.001 | 255 | 1.63 (1.22~2.17) | <0.001 | 544 | 1.52 (1.32~1.79) | <0.001 |
| High hs-CRP alone | 99 | 48 | 1.47 (1.11~2.01) | <0.001 | 22 | 1.95 (1.24~3.25) | 0.001 | 26 | 1.45 (0.98~2.17) | 0.065 |
| Vitamin D deficiency + high hs-CRP | 346 | 184 | 2.07 (1.51~2.34) | <0.001 | 71 | 1.87 (1.28~2.739) | <0.001 | 113 | 2.14 (1.57~2.68) | <0.001 |
| RERI (95%CI) |  |  | 0.05 (-0.03~0.13) |  |  | -0.71 (-1.65~0.23) |  |  | 0.17 (-0.09~0.43) |  |

Adjusting for age, sex, province of sampling, the month of sampling, ethnicity, residence, living status, education, marital status, outdoor activities, vitamin supplements, smoking, drinking, BMI, ADL impairment, cognitive function, depression, albumin, blood glucose, cholesterol, triglycerides, creatinine, medical care treatment, hypertension, cerebrovascular disease, respiratory disease, and cancer. 25(OH)D, 25-dihydroxyvitamin D; hs-CRP, high sensitivity C-reactive protein; CVD, cardiovascular disease; HR, hazard ratio; CI, confidence interval; RERI, relative excess risk due to interaction.

**Supplementary Table 3. Sensitivity analysis of combined effects of 25(OH)D deficiency and high hs-CRP on mortality.**

**Excluding participants with cerebrovascular disease, respiratory disease, or cancer at baseline.**

| Groups | Participants | **All-cause mortality** | | | **CVD mortality** | | | **Non-CVD mortality** | | |
| --- | --- | --- | --- | --- | --- | --- | --- | --- | --- | --- |
|  |  | Deaths | Adj-HRs (95%CI) | *P* value | Deaths | Adj-HRs (95%CI) | *P* value | Deaths | Adj-HRs (95%CI) | *P* value |
| **Panel A:** |  |  |  |  |  |  |  |  |  |  |
| **Excluding cerebrovascular disease (n=2823)** |  |  |  |  |  |  |  |  |  |  |
| Vitamin D sufficiency + normal hs-CRP | 619 | 176 | Ref. | — | 55 | Ref. | — | 121 | Ref. | — |
| Vitamin D deficiency alone | 1615 | 726 | 1.50 (1.18~1.85) | <0.001 | 228 | 1.68 (1.24~2.29) | <0.001 | 498 | 1.63 (1.41~2.11) | <0.001 |
| High hs-CRP alone | 135 | 64 | 1.46 (1.09~1.95) | <0.001 | 26 | 1.87 (1.22~3.01) | <0.001 | 38 | 1.52 (1.04~2.22) | 0.031 |
| Vitamin D deficiency + high hs-CRP | 454 | 248 | 2.13 (1.66~2.47) | <0.001 | 98 | 2.34 (1.65~3.30) | <0.001 | 150 | 2.02 (1.57~2.61) | <0.001 |
| RERI (95%CI) |  |  | 0.16 (0.02~0.29) |  |  | -0.20 (-0.59~0.18) |  |  | -0.13 (-0.36~0.08) |  |
| **Panel B:** |  |  |  |  |  |  |  |  |  |  |
| **Excluding respiratory disease (n=2788)** |  |  |  |  |  |  |  |  |  |  |
| Vitamin D sufficiency + normal hs-CRP | 597 | 161 | Ref. | — | 53 | Ref. | — | 108 | Ref. | — |
| Vitamin D deficiency alone | 1624 | 733 | 1.61 (1.19~1.96) | <0.001 | 232 | 1.55 (1.15~2.06) | <0.001 | 501 | 1.71 (1.49~2.02) | <0.001 |
| High hs-CRP alone | 121 | 52 | 1.44 (1.09~1.97) | 0.001 | 19 | 1.67 (1.19~3.11) | <0.001 | 33 | 1.55 (1.04~2.32) | 0.018 |
| Vitamin D deficiency + high hs-CRP | 446 | 241 | 2.14 (1.56~2.42) | <0.001 | 93 | 2.39 (1.64~3.49) | <0.001 | 148 | 1.93 (1.42~2.41) | <0.001 |
| RERI (95%CI) |  |  | 0.09 (-0.02~0.21) |  |  | 0.17 (-0.03~0.37) |  |  | -0.34 (-1.25~0.58) |  |
| **Panel C:** |  |  |  |  |  |  |  |  |  |  |
| **Excluding cancer (n=2915)** |  |  |  |  |  |  |  |  |  |  |
| Vitamin D sufficiency + normal hs-CRP | 634 | 173 | Ref. | — | 58 | Ref. | — | 115 | Ref. | — |
| Vitamin D deficiency alone | 1667 | 758 | 1.58 (1.25~1.95) | <0.001 | 242 | 1.64 (1.21~2.22) | <0.001 | 516 | 1.75 (1.52~2.27) | <0.001 |
| High hs-CRP alone | 137 | 63 | 1.50 (1.12~2.00) | 0.001 | 24 | 1.69 (1.12~2.73) | 0.003 | 39 | 1.51 (1.05~2.22) | 0.029 |
| Vitamin D deficiency + high hs-CRP | 477 | 261 | 2.23 (1.75~2.59) | <0.001 | 103 | 2.39 (1.68~3.36) | <0.001 | 159 | 2.08 (1.61~2.68) | <0.001 |
| RERI (95%CI) |  |  | 0.15 (0.03~0.26) |  |  | 0.05 (-0.19~0.29) |  |  | -0.19 (-0.51~0.11) |  |

Adjusting for age, sex, province of sampling, the month of sampling, ethnicity, residence, living status, education, marital status, outdoor activities, vitamin supplements, smoking, drinking, BMI, ADL impairment, cognitive function, depression, albumin, blood glucose, cholesterol, triglycerides, creatinine, medical care treatment, hypertension, cerebrovascular disease (not in panel A), respiratory disease (not in panel B), and cancer (not in panel C). 25(OH)D, 25-dihydroxyvitamin D; hs-CRP, high sensitivity C-reactive protein; CVD, cardiovascular disease; HR, hazard ratio; CI, confidence interval; RERI, relative excess risk due to interaction.

**Supplementary Table 4.** **Subgroup analysis of combined effects of 25(OH)D deficiency and high hs-CRP on mortality stratified by medical care treatment**

| Groups | Participants | **All-cause mortality** | | | **CVD mortality** | | | **Non-CVD mortality** | | |
| --- | --- | --- | --- | --- | --- | --- | --- | --- | --- | --- |
|  |  | Deaths | Adj-HRs (95%CI) | *P* value | Deaths | Adj-HRs (95%CI) | *P* value | Deaths | Adj-HRs (95%CI) | *P* value |
| **Medical care treatment (n=1411)** |  |  |  |  |  |  |  |  |  |  |
| Vitamin D sufficiency + normal hs-CRP | 336 | 88 | Ref. | — | 25 | Ref. | — | 63 | Ref. | — |
| Vitamin D deficiency alone | 814 | 276 | 1.37 (1.08~1.51) | 0.027 | 69 | 1.17 (0.89~1.58) | 0.215 | 207 | 1.42 (1.23~1.62) | 0.015 |
| High hs-CRP alone | 46 | 15 | 1.26 (0.95~1.71) | 0.131 | 3 | 1.23 (0.77~1.79) | 0.397 | 12 | 1.41 (0.97~2.04) | 0.096 |
| Vitamin D deficiency + high hs-CRP | 215 | 81 | 1.85 (1.46~2.17) | <0.001 | 28 | 1.83 (1.31~2.54) | 0.001 | 53 | 1.79 (1.47~2.39) | <0.001 |
| RERI (95%CI) |  |  | 0.24 (0.25~0.72) |  |  | 0.42 (-79~1.63) |  |  | -0.03 (-1.02~0.96) |  |
| **Non-medical care treatment (n=1661)** |  |  |  |  |  |  |  |  |  |  |
| Vitamin D sufficiency + normal hs-CRP | 336 | 97 | Ref. | — | 35 | Ref. | — | 62 | Ref. | — |
| Vitamin D deficiency alone | 942 | 523 | 1.81 (1.45~2.01) | <0.001 | 186 | 1.89 (1.45~2.56) | <0.001 | 337 | 1.74 (1.51~1.98) | <0.001 |
| High hs-CRP alone | 96 | 50 | 1.61 (1.21~2.17) | <0.001 | 23 | 2.18 (1.36~3.19) | <0.001 | 27 | 1.61 (1.11~2.33) | 0.008 |
| Vitamin D deficiency + high hs-CRP | 287 | 191 | 2.58 (2.04~3.03) | <0.001 | 79 | 2.25 (1.68~3.27) | <0.001 | 112 | 2.28 (1.86~3.03) | <0.001 |
| RERI (95%CI) |  |  | 0.16 (0.02~0.31) |  |  | -0.72 (-2.01~0.55) |  |  | -0.06 (-0.18~0.05) |  |

Adjusting for age, sex, province of sampling, the month of sampling, ethnicity, residence, living status, education, marital status, outdoor activities, vitamin supplements, smoking, drinking, BMI, ADL impairment, cognitive function, depression, albumin, blood glucose, cholesterol, triglycerides, creatinine, medical care treatment, hypertension, cerebrovascular disease, respiratory disease, and cancer. 25(OH)D, 25-dihydroxyvitamin D; hs-CRP, high sensitivity C-reactive protein; CVD, cardiovascular disease; HR, hazard ratio; CI, confidence interval; RERI, relative excess risk due to interaction.

*P*-interactions of medical care treatment and combined vitamin D deficiency/ high hs-CRP on all-cause mortality, CVD mortality, and non-CVD mortality were 0.204, 0.168, and 0.239.

**Supplementary Table 5. Associations of 25(OH)D with mortality risk after excluding 169 participants who frequently supplemented with vitamins**

| **Groups** | **Participants, n** | **Deaths, n%** | **Adj-HR (95% CI)** |
| --- | --- | --- | --- |
| **Serum 25(OH)D** |  |  |  |
| **All-cause mortality** |  |  |  |
| ≧50.71 nmol/L | 715 | 218 (30.49) | Ref. |
| ≧36.88 and <50.71 nmol/L | 729 | 273 (37.45) | 1.21 (1.02~1.45) |
| ≧26.72 and <36.88 nmol/L | 726 | 344 (47.38) | 1.36 (1.12~1.62) |
| < 26.72 nmol/L | 733 | 414 (56.48) | 1.53 (1.28~1.79) |
| *P*-trend |  | <0.001 | <0.001 |
| **CVD mortality** |  |  |  |
| ≧50.71 nmol/L | 715 | 77 (10.77) | Ref. |
| ≧36.88 and <50.71 nmol/L | 729 | 96 (13.17) | 1.22 (0.95~1.77) |
| ≧26.72 and <36.88 nmol/L | 726 | 125 (17.22) | 1.36 (1.03~1.81) |
| < 26.72 nmol/L | 733 | 129 (17.60) | 1.39 (1.02~1.98) |
| *P*-trend |  | 0.019 | 0.031 |
| **Non-CVD mortality** |  |  |  |
| ≧50.71 nmol/L | 715 | 141 (19.72) | Ref. |
| ≧36.88 and <50.71 nmol/L | 729 | 177 (24.28) | 1.22 (0.97~1.51) |
| ≧26.72 and <36.88 nmol/L | 726 | 219 (30.17) | 1.43 (1.17~1.77) |
| < 26.72 nmol/L | 733 | 285 (38.88) | 1.82 (1.49~2.31) |
| *P-*trend |  |  | <0.001 |

Adjusting for age, sex, province of sampling, the month of sampling, ethnicity, residence, living status, education, marital status, outdoor activities; smoking, drinking; ADL, cognitive function, depression, albumin, blood glucose, cholesterol, triglycerides, creatinine, medical care treatment, hypertension, cerebrovascular disease, respiratory disease, cancer, and hs-CRP. 25(OH)D, 25-dihydroxyvitamin D; CVD, cardiovascular disease; HR, hazard ratios; CI, confidence interval.

**S****upplementary Table 6. Combined associations of 25(OH)D deficiency and high hs-CRP with mortality risk after additionally adjusting for high-density lipoprotein and low-density lipoprotein**

| **Groups** | **Participants** | **All-cause mortality** | | | **CVD mortality** | | | **Non-CVD mortality** | | |
| --- | --- | --- | --- | --- | --- | --- | --- | --- | --- | --- |
|  |  | Deaths | Adj-HRs (95%CI) | *P* value | Deaths | Adj-HRs (95%CI) | *P* value | Deaths | Adj-HRs (95%CI) | *P* value |
| Vitamin D sufficiency + normal hs-CRP | 672 | 185 | Ref. | — | 60 | Ref. | — | 125 | Ref. | — |
| Vitamin D deficiency alone | 1756 | 799 | 1.47 (1.24~1.73) | <0.001 | 255 | 1.58 (1.19~1.99) | 0.007 | 544 | 1.50 (1.23~1.84) | <0.001 |
| High hs-CRP alone | 142 | 65 | 1.55 (1.17~2.01) | 0.002 | 26 | 1.91 (1.19~3.06) | 0.006 | 39 | 1.46 (1.02~2.11) | 0.040 |
| Vitamin D deficiency + high hs-CRP | 502 | 272 | 2.21 (1.79~2.64) | <0.001 | 107 | 2.36 (1.59~3.13) | 0.001 | 165 | 2.24 (1.71~2.61) | <0.001 |
| RERI (95%CI) |  |  | 0.24 (0.03~0.44) |  |  | -0.13 (-0.33~0.08) |  |  | 0.28 (0.07~0.48) |  |

Adjusting for age, sex, province of sampling, the month of sampling, ethnicity, residence, living status, education, marital status, outdoor activities, vitamin supplements, smoking, drinking, BMI, ADL impairment, cognitive function, depression, albumin, blood glucose, cholesterol, triglycerides, creatinine, medical care treatment, hypertension, cerebrovascular disease, respiratory disease, cancer, high-density lipoprotein, and low-density lipoprotein. 25(OH)D, 25-dihydroxyvitamin D; hs-CRP, high sensitivity C-reactive protein; CVD, cardiovascular disease; HR, hazard ratio; CI, confidence interval; RERI, relative excess risk due to interaction.
